# New Advances in Alzheimer's Disease: From Biology to Therapy

**DOI:** 10.2174/138920207783769558

**Published:** 2007-12

**Authors:** Giuseppina Tesco

Alzheimer's disease (AD) is a devastating neurodegenerative disorder that results in the loss of memory and cognitive function, and eventually dementia. AD is the most common cause of dementia and the eighth leading cause of death. Increasing age is the greatest risk factor for AD. Under the age of 65 the disease is rare, but it increases dramatically: the disease affects one in 10 individuals over age of 65 and nearly half the population over age of 85. Some 4.5 million individuals in the United States have Alzheimer's disease [1, 2]. Scientists estimate that as the population continues to age and baby boomers reach retirement age and beyond, this number will triple by the year 2050. With this expected increase in the prevalence of AD there will be an increase in the financial burden caused by this condition. Thus, therapies aimed at the prevention and the treatments of AD are desperately needed. This issue of Current Genomics is dedicated to the most recent advances in AD research, which began 100 years ago when the disease was first described.

A key neuropathological event in AD is the cerebral accumulation of an ~4kDa peptide termed Aβ, the principle component of senile plaques. Amyloid plaques are formed by aggregates of amyloid-β-peptides, 37-43 amino-acid fragments (predominantly Aβ_40_ and Aβ_42_) derived by serial proteolysis of the amyloid precursor protein (APP) by β- and γ-secretase. APP more commonly undergoes a non-amyloidogenic processing by α-secretase that cleaves in the middle of the β-amyloid domain [3]. β-secretase has been identified as a novel membrane-tethered member of the aspartyl proteases, termed BACE [4, 5], while candidate α-secretases include ADAM 9, 10 and 17 (TACE, tumor necrosis factor-α converting enzyme) [6, 7]. Recent findings have shown that γ-secretase is a complex of four proteins including presenilin, nicastrin/Aph2, Aph1 and Pen-2 [8]. 

A central role for Aβ in AD pathogenesis is supported by studies of human genetics and data derived from both *in vitro* and *in vivo* models. In this issue of Current Genomics, Dr. Lacor reviews the findings that had led to development of the β-amyloid hypothesis, which states that Aβ accumulation is the initial step of a cascade of events starting with dysfunction of synaptic transmission and evolving in the degeneration of neuronal cells. Aβ can exist in a variety of different forms, including monomers, oligomers, protofibrils and fibrils. Dr. Lacor clearly discusses the toxicity of the different Aβ forms focusing on the synaptic toxicity of oligomeric species. These soluble toxins would account for the poor correlation between the number of amyloid plaques and disease progression, and could provide a unifying mechanism for AD pathogenesis. 

Since β-secretase processing of APP is the initial step of Aβ generation, BACE is an important target for therapeutic intervention. Drs. Cole and Vassar, who discovered BACE in 1999, review in this issue the biology of BACE with emphasis on its role in normal and disease conditions. Increasing evidence show that BACE is a stress-induced protease, which is upregulated in the brains of AD subjects, following experimental stroke and head trauma among other conditions. In addition to APP, other transmembrane proteins are processed by BACE raising the question of whether BACE inhibition could lead to serious side effects. The fact that BACE null mice are viable and fertile, and are apparently normal, at least early in life, suggests that the inhibition of BACE still represents a primary target for the treatment of AD. 

Gamma-secretase cleavage follows β-secretase processing of APP and results in the production of Aβ. The inhibition of γ-secretase is another important target for anti-Aβ therapies. Gamma secretase activity requires the assembly of four proteins including presenilin, nicastrin/Aph2, Aph1 and Pen-2. In this issue Dr. Fraering reviews the role of each components in the regulation of γ-secretase activity. Furthermore, he discusses the most recent findings on the structure of γ-secretase. Electron microscopy (EM) and single particle image analysis of purified  γ-secretase complex revealed a globular structure with a low-density central (and possibly water-containing) intramembrane chamber. Further structural studies are required to fully understand how γ-secretase cleaves its substrates within the transmembrane domain. These studies will also help to develop γ-secretase inhibitors or modulators able to prevent Aβ generation without affecting the processing of other vital γ-secretase substrates [9]. 

Dr. Lleo’s article reviews the therapies currently available to the clinical neurologist for the treatment of subjects affected by AD. Unfortunately these treatments, acetylcholinesterase inhibitors and memantine, an NMDA antagonist, have only modest effects on the symptoms without affecting the progression of the disease. However, the advances in basic research reviewed in this special issue may facilitate the development of more effective drugs e.g. BACE and γ-secretase inhibitors as well as therapies aimed to increase the clearance of Aβ toxic species.
